# An observational study of the effectiveness of practice guideline implementation strategies examined according to physicians' cognitive styles

**DOI:** 10.1186/1748-5908-2-41

**Published:** 2007-12-01

**Authors:** Lee A Green, Leon Wyszewianski, Julie C Lowery, Christine P Kowalski, Sarah L Krein

**Affiliations:** 1 Department of Family Medicine, Medical School, University of Michigan, Ann Arbor, Michigan, USA; 2 Department of Health Management & Policy, School of Public Health, University of Michigan Ann Arbor, Michigan, USA; 3 Health Services Research & Development, Ann Arbor Veterans Administration Hospital and Health Center, Ann Arbor, Michigan, USA; 4 Department of Internal Medicine, University of Michigan Medical School, Ann Arbor, Michigan, USA

## Abstract

**Background:**

Reviews of guideline implementation recommend matching strategies to the specific setting, but provide little specific guidance about how to do so. We hypothesized that the highest level of guideline-concordant care would be achieved where implementation strategies fit well with physicians' cognitive styles.

**Methods:**

We conducted an observational study of the implementation of guidelines for hypertension management among patients with diabetes at 43 Veterans' Health Administration medical center primary care clinics. Clinic leaders provided information about all implementation strategies employed at their sites. Guidelines implementation strategies were classified as education, motivation/incentive, or barrier reduction using a pre-specified system. Physician's cognitive styles were measured on three scales: evidence vs. experience as the basis of knowledge, sensitivity to pragmatic concerns, and conformity to local practices. Doctors' decisions were designated guideline-concordant if the patient's blood pressure was within goal range, or if the blood pressure was out of range and a dose change or medication change was initiated, or if the patient was already using medications from three classes.

**Results:**

The final sample included 163 physicians and 1,174 patients. All of the participating sites used one or more educational approaches to implement the guidelines. Over 90% of the sites also provided group or individual feedback on physician performance on the guidelines, and over 75% implemented some type of reminder system. A minority of sites used monetary incentives, penalties, or barrier reduction. The only type of intervention that was associated with increased guideline-concordant care in a logistic model was barrier reduction (p < 0.02). The interaction between physicians' conformity scale scores and the effect of barrier reduction was significant (p < 0.05); physicians ranking lower on the conformity scale responded more to barrier reduction.

**Conclusion:**

Guidelines implementation strategies that were designed to reduce physician time pressure and task complexity were the only ones that improved performance. Education may have been necessary but was clearly not sufficient, and more was not better. Incentives had no discernible effect. Measurable physician characteristics strongly affected response to implementation strategies.

## Background

Reviews of research on practice guidelines implementation [1,2] and physician practice change [3-7] now widely conclude that no one type of intervention is likely to be successful, and that implementation efforts should use a combination of strategies tailored to the setting. At present no concrete guidance is available regarding how to match tools to settings. Indeed, the entire field of practice change interventions is deficient in theoretical grounding and in critical evaluation [8,9], making it difficult to predict whether interventions will succeed or even to understand why they worked or failed in any given trial. However, critics of calls for more theoretical grounding have pointed out that, while theoretical guidance is desirable in theory, empirical evidence of its usefulness is lacking [10].

We sought to empirically test a theory-based approach to choosing guideline implementation strategies, based on the hypotheses that individual variation is important and the fit between individual and strategy is a key determinant of success. Previously, we developed a typology of cognitive styles, postulating that there are four archetypes of physician response patterns to new information intended to change practice [11]. These four are the "seeker", strongly evidence-based and willing to act on evidence almost regardless of other factors; the "receptive", who regards data as the basis of knowledge but attends also to setting and social issues; the "traditionalist", who regards clinical experience and authority rather than data as the basis of knowledge; and the "pragmatist", who is less concerned about the basis of knowledge than about the practicalities of getting patients seen. This typology is based on three underlying psychometric scales: evidence vs. experience orientation as the basis of knowledge ("E"), sensitivity to pragmatic concerns such as time and patient flow ("P"), and conformity to local practices and group norms ("C"). We have published a measurement instrument for these scales [12], which we hereafter term the "EPC instrument."

In 1995 the Department of Veterans' Affairs (VA) health system began a system-wide re-engineering of its clinics. As part of that process, formal practice guidelines for several high-priority conditions were developed and disseminated. The guidelines were developed centrally, but each local site had wide latitude in choosing strategies for implementing them, and the resulting variation in implementation methods of a common guideline provided a large-scale natural experiment. We conducted an observational cohort study of the VA system's implementation of guidelines for hypertension among patients with diabetes, hypothesizing that the fit between physicians' measured cognitive styles on the EPC instrument and sites' chosen implementation strategies would predict guideline-concordant practice.

## Methods

This multi-site study collected data at three levels: site, physician, and patient.

### Site level

We approached hospital directors at 59 VA Medical Centers (VAMCs), and 43 agreed to have their facility participate in the study. The participating VAMCs are located in 27 states, and in 19 of the 21 Veterans Integrated Service Networks (VISNs). Semi-structured telephone interviews were conducted with two key informants at each of the participating VAMCs. These informants' roles were Chiefs of Staff, Associate Chiefs of Staff for Ambulatory Care, Quality Managers, or Directors of Primary Care. Interviewees were asked to answer questions in relation to the period between 1999 – when revised VA hypertension guidelines were published – and 2001, when the interviews for the study began. Interview respondents were asked to describe all steps taken to implement guidelines for hypertension management in patients with Type 2 diabetes at their VAMC's primary care clinics. A total of 86 interviews were conducted from July 2001 to August 2002.

The transcribed notes from the interviews, describing in detail the guideline implementation interventions used at each site, were coded For each participating site, the number of interventions in each of 27 categories was recorded. The 27 categories were derived from a pre-specified framework (available from the authors upon request), that distinguishes between three classes of interventions: education (*e.g*., evidence-based lectures); motivation/incentives (*e.g*., individual performance feedback); and barrier-reduction (*e.g*., freeing physician time to discuss treatment by reassigning other tasks to support staff). The delineation of these categories draws on earlier formulations [5,13-15] and parallels the framework of Cabana *et al*. [4].

### Physician level

IRB approval for the physician data and the patient data phases was a time-consuming process that lasted approximately 19 months and eventually resulted in approval from 42 of the 43 medical centers (representing 18 of the 21 VISNs) participating in the site interviews [16].

**Figure 1:**
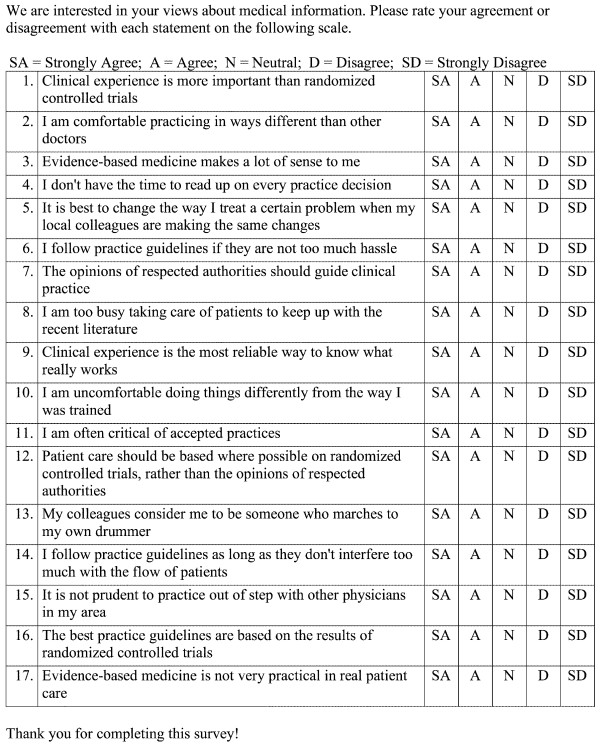
EPC Instrument.

At the physician level, consenting physicians at each site completed the same one-page 17-item questionnaire (Figure 1, the EPC instrument) on two occasions. The questionnaire is designed to measure the three scales (E, P, and C) described above [12]. The scale scores subsequently form the basis for classifying physicians into the four archetypal categories previously defined: seeker, receptive, traditionalist, and pragmatist.

The questionnaire was mailed to all primary care physicians (PCPs) at the participating medical centers between June 2002 and December 2003. A second mailing was sent to each participating physician one year after the first questionnaire, to assess test-retest reliability and confirm that the scales measure a stable characteristic.

Principal components factor analysis with orthogonal varimax rotation was performed on the responses to the first questionnaire. The eigenvalues from the factor analysis were used to determine the number of factors in the optimum solution. The instrument's questions were then assigned to these factors based upon which factor they loaded most heavily on in the rotated solution. This analysis was identical to that in the instrument's validation [12], and was used to confirm the scales. In addition, questionnaire responses were used to assign each physician to one of the four types (seeker, receptive, traditionalist, or pragmatist). First and second year questionnaire responses within physician were compared using Pearson correlation statistics.

### Patient data level

To measure concordance between physicians' prescribing and guideline recommendations for diabetes patients with hypertension, the diabetes cohort was defined as all patients at the 42 sites who had filled a prescription for diabetes medications or blood glucose monitoring supplies; or had one inpatient or two outpatient encounters with a diabetes related ICD-9 code (250.x, 357.2, 362.0–362.1, 366.41) in fiscal year (FY) 1999. Patient-level data on antihypertensive prescriptions (including prescribing provider), outpatient visits, and blood pressures were obtained on these patients from VA national datasets for 1999 and 2000 (considered post-guideline implementation). Patients were assigned to a specific PCP if more than 50% of their outpatient medical clinic visits (excluding visits for psychiatric or ancillary services) during FY 1999 were with that PCP. These data were then merged with our cohort of PCPs who returned their surveys to limit the database to only those diabetes patients who had participating PCPs. Finally, only patients with blood pressure data during the period 1999–2000 were included in the analysis.

The outcome variable was blood pressure at goal or appropriate physician decision making for blood pressures not at goal. Specifically, patients were identified whose last blood pressure reading in the first 18 months of the study period (1999–2000) was elevated (systolic ≥ 140 or diastolic ≥ 90, the guideline criterion at the time). An indicator variable was created, with a value of one assigned if management was not consistent with the guideline and zero otherwise. Guideline-consistent care was defined using a "tightly linked" measure, *i.e*., a measure that focuses on processes of care whose link to blood pressure has been clearly established by scientific evidence [17]. Specifically, following an elevated blood pressure reading, patients' management was considered guideline consistent if any one or more of the following criteria were met:

1. Already on three or more blood pressure medication classes. Blood pressure medications were grouped into classes as follows: thiazide diuretics, ACE inhibitors, beta blockers, calcium channel blockers, alpha blockers, and angiotensin II inhibitors. Data on prescriptions of centrally-acting agents (*e.g*., reserpine) were not available.

2. Having an increase in medication dose during the 6 months following the elevated reading

3. Having another medication class added or medication class switch during the 6 months following the elevated reading

4. Having a repeat blood pressure reading of <140/90 mmhg during the 6 months following the elevated reading

### Analysis

Concordance scores were constructed for each physician to quantify the extent to which the interventions implemented at their sites were suited, according to our framework, to their specific physician type as measured by the EPC instrument. The scores are based on a table of weights [see Additional file 1] ranging from -1 to 5, quantifying the relationship between physician type (one of the four categories determined from the physician questionnaire) and intervention as hypothesized by Green and Wyszewianski [18]. Each weight indicates the degree to which that type of intervention is hypothesized to be likely to improve guideline adherence for that type of physician; 0 is a complete lack of effect and -1 represents a counterproductive effect. The scores were developed by the authors based on the theory of physician types and on the existing practice change literature (for example, information provided by local opinion leaders is expected to be more effective than information from others). The concordance score for a physician was the sum of concordance sub-scores for that physician's type for each intervention implemented at the physician's medical center. Scores were summed, not averaged, within physician. This approach was chosen as we deemed it likely that sites using larger numbers of interventions would have greater effects, though the choice of the linear arithmetic sum rather than a diminishing-returns curve was arbitrary.

The primary hypothesis was tested in a logistic model with concordance score as the independent variable, and correction for correlations among patients by physician, using STATA's [19] clustered logistic regression algorithm. Logistic regression using a more detailed model was then carried out. All three scales (E, P, and C) were retained as independent variables throughout this secondary modeling. For each class of guideline implementation intervention (educational, motivation-oriented, and barrier-reduction), the number of interventions that was used at each site was also entered as an independent variable. Then, the interaction terms between intervention classes and scales were entered. The intervention counts and interaction terms were retained only if their *p *< 0.1 (|z| >1.65) in a forward-stepping Wald procedure. Lastly, the specific effects of individual and group incentives, penalties, and feedback to physicians (the six kinds of interventions that made up the motivational class) were tested in the same model by entering the numbers of each of those at each site, again using the forward-stepping Wald procedure, to test the possibility that different kinds of motivational interventions might have effects different from the overall motivational class effect.

## Results

Table 1 shows the numbers of patients and primary care physicians (PCPs) in the sample. The average number of qualifying patients/PCP was seven, with a range of one to 47. The average age of patients in the sample was 65.1 (s.d. = 11.4, range 25.5 – 88.3). Most were male (97.3%) and white (67.0%). The 163 PCPs in the final sample represent 22% of the total number of PCPs at the 42 sites, and the 1174 patients represents 0.6 % of the cohort of diabetes patients for these sites.

**Table 1:** Study Cohort Derivation

Phase	Primary Physicians	Patients
Initial Recruitment at 42 sites	291 usable surveys	208,653 diabetes patients
Matching of patients to PCPs	185	1875
Blood pressure data available	163	1174

### Site level

Results from interviews showed that all of the participating sites used one or more educational intervention(s) to implement the guidelines, including distribution of written materials, didactic presentations, and interactive conferences. The mean number of education interventions was three, with a maximum of seven. Motivational interventions were the next most prevalent class; in particular, over 90% of the sites provided group, individual, or both group and individual feedback on physician performance on the guidelines, while monetary incentives or penalties were seldom used. Barrier reduction was the least-used class, with fewer than 50% of sites undertaking any barrier-reduction strategy.

Qualitatively, time pressure was an overarching theme at the site level. 72% of the sites spontaneously (*i.e*., without prompting) mentioned the challenges associated with adhering to practice guidelines given the time and workload pressures in their clinics. These perceptions are quantitatively supported by VA workload data, which show that the number of primary care visits increased by 31% from 7.1 million in 1998 to 9.3 million in 2001 (the time period immediately prior to and during the study period) [20].

### Physician level

Of 745 questionnaires distributed to primary care physicians, 307 were returned (response rate of 41.2%). Of the 307 questionnaires returned, 16 had missing data, leaving the 291 usable questionnaires listed in Table 1.

Factor analysis generally confirmed the 3-factor psychometric scaling used previously. Question ten did not load cleanly, and inspection revealed that it dealt with past training not current practice; so, it was dropped. Question seven loaded equally on the E and C scales, and hence was not useable.

The physicians in this sample tended toward an evidence-based orientation: the mean score on the E scale, which spans from 5 to 30, was 24.1 (range 17 – 30). In addition, this sample consisted primarily of pragmatists, as we have observed in other community physician samples. According to our physician classification system [11], there were 174 pragmatists (59.8%), 80 receptives (27.5%), 36 seekers (12.4%), and 1 traditionalist (0.3%). Of the 291 participating providers with useable surveys, 263 (90%) completed follow-up surveys one year later. The correlations for the E, P, and C subscales were 0.75, 0.68, and 0.75 respectively.

The mean concordance score was 17.9 (SD = 7.76, range 4 – 36), indicating a high level of guidelines implementation activity with a broad range of concordance (from good fit to poor) across sites.

### Patient level

Overall, decisions tended to adhere to the guideline: 77.2% of patients received guideline-consistent care as defined by the four criteria above. Across sites, the range of guideline-consistent care was 50% to 100%, with a standard deviation of 13%. Across physicians, the range was 0 to 100% with a standard deviation of 28%.

The initial hypothesis was not supported: there was no association between concordance score and guideline-consistent decision making. The odds ratio for the effect of concordance score on guideline consistent care was 0.99, p = 0.40.

**Figure 2:**
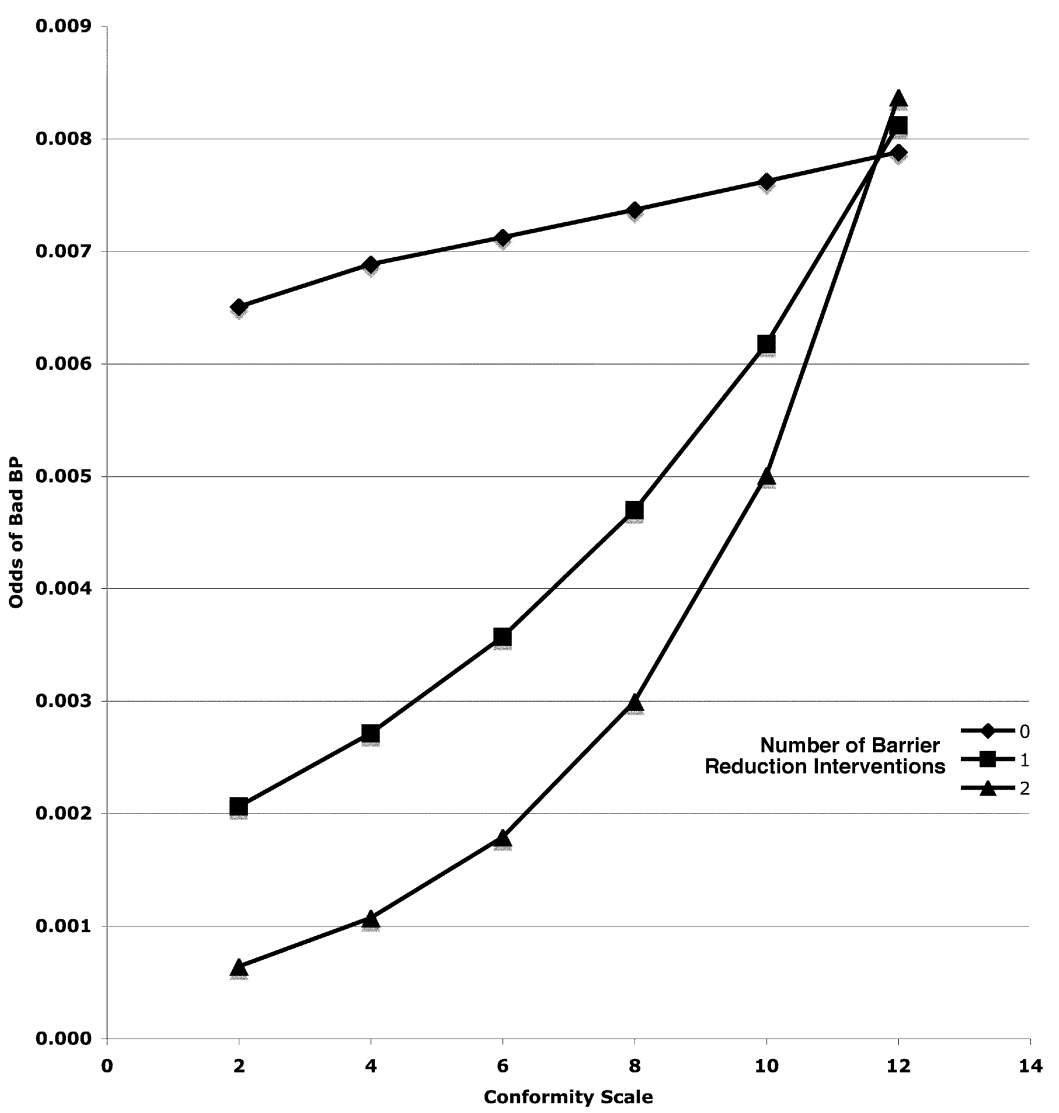
**Odds of Guideline-Nonconcordant Blood Pressure Management by Physician Conformity Scale Score**. Sites Implementing 0, 1, or 2 (triangle) Barrier Reduction Strategies

In the detailed logistic modeling, of the three EPC scales only the C scale predicted guideline-consistent care (lower conformity associated with better decisions, p < 0.05). None of the three types of interventions had an effect on guideline concordance. When interaction terms were introduced, the only type of intervention that was associated with guideline-consistent care was barrier reduction (p < 0.02). The C scale had no independent effect when interactions were included: the interaction between C scale score and barrier reduction was significant (p < 0.05), with the least conformity-oriented physicians improving most with barrier reduction (Figure 2). Incentives, penalties, and feedback had no measurable effects.

## Discussion

This empirical test of an implementation theory was partially successful. The theory itself was not supported: having an implementation strategy that matched physician style did not generally predict outcome. However, the application of the theory did provide some explanation of the mechanism and pattern of implementation success and failure that may be useful in further research.

This observational study of a natural experiment also provided a simultaneous trial of most of the currently advocated implementation strategies. It was a negative trial of education, audit and feedback, incentives, and clinical reminders. Barrier reduction interventions were successful but only for a subgroup of physicians, and the theory-directed EPC instrument identified that subgroup.

Barrier reduction strategies, *i.e*., guideline implementation strategies that were designed to reduce the effort or complexity of a task, were the only ones associated with better performance in this setting. The interaction between psychometric scale C and implementation strategy showed that the best performing combination was physicians willing to practice differently from the local norm in settings where barrier reduction was undertaken. More conformity-oriented physicians did not do better in reduced-barrier settings; they may require more compulsory interventions, more social support, or more peer pressure.

Education may have been necessary but was clearly not sufficient: all sites included education in their mix of strategies, but those doing a great deal of it saw no more effect than those doing the minimum.

A strong belief in evidence did not affect performance, nor did general sensitivity to pragmatic concerns (time, workflow, and patient acceptance). The latter finding may seem surprising, given the frequency with which time pressure concerns were expressed by physicians. It is important to understand that the P scale measures trait sensitivity to pragmatic concerns, not state; that is, it does not assess how affected the physician currently is by pragmatic concerns, but rather how they believe such concerns should affect practice. In daily clinical operations, most physicians must act in accordance with pragmatic concerns most of the time, but those concerns may not be the basis on which they respond to practice change interventions.

Hysong *et al*. [21], in a qualitative study in the VA system, found that high- and low-performing sites with respect to guideline concordant care carried out audit and feedback interventions differently. It is possible that the overall negative results we observed with most of the guideline implementation strategies reflect a mix of effective and ineffective applications of those strategies.

These findings were observed in a system where time and efficiency pressures are very high, where essentially all slack has been squeezed out. Different patterns might well be found in less pressured settings. For example, if they had a small amount of free time to work with, more physicians may have responded to educational and incentive interventions even when the system did not change to enable such responses.

The high baseline rate of guideline-consistent care may have also affected the results we observed. With most physicians in our sample already using multiple medications to treat high blood pressure in this group of diabetes patients, the opportunity for interventions to show an effect could have been limited. Both time pressure and high baseline care quality may have prevented improvement from incentives: with appropriate care already prevalent, the "low-hanging fruit" was probably already picked, leaving only the most difficult improvements remaining, and incentives may not have been able to overcome system barriers to achieve them. Greater variation in guideline adherence between sites and between physicians might have permitted a larger effect to emerge in the data, but the variation in this sample was probably sufficient to demonstrate large enough effects to be operationally useful.

Other possible contributors to the observed findings are limitations in the study data. A major limitation is the small sample size of physicians (163) in relationship to the number of physicians who were asked to participate in the study (745). The sample size of patients was significantly reduced from the total number of diabetes patients in our participating sites because of the inability to match patients with providers. Even though all patients in the VAMCs are supposed to have a primary care provider, it was still challenging to meet the criterion that more than 50% of a patient's outpatient medical clinic visits had to be to one of our participating PCPs. There was also physician turnover during the interval between the patient visits and the questionnaires. Further, missing blood pressure data eliminated additional patients from the sample.

However, the majority of the reduction in sample size was due to physicians not agreeing to participate. We do not know with certainty why the rate was so low; it could be due to physicians not willing to accept the potential risks of participation or to their unwillingness to take the time to complete the survey. However, the risk was very low and the survey was a single page taking only a few minutes to complete. We suspect that participation was discouraged by the daunting nature of the consent forms required, which ranged from three to seven pages [16]. Other studies conducted at our center that have not required written consent forms for similar surveys have attained considerably higher participation rates by providers, and our validation studies using the same survey have experienced no difficulty with recruitment.

A sample bias in favor of compliance with guidelines might be hypothesized on the basis of physician self-selection for response and because patients who have good PCP continuity relationships may adhere better to treatment. However, Petersen *et al*. found similarly high rates of appropriate care in a sample of over 237,000 VA patients in 2004–2005, suggesting that our findings were not unrepresentative [22].

Test-retest correlation supports the belief that the scales are relatively stable characteristics of physicians. We do not know whether they might alter with change of setting but they seem to be consistent over time within settings.

## Conclusion

We found that implementation success was associated with measurable physician traits interacting with implementation strategy, and that a theory-based study could improve our ability to understand success and failure of implementation.

These results suggest that efforts to improve adherence to practice guidelines (and other evidence-based practice recommendations) should focus on barrier reduction in organized primary care settings where time pressure is high. That is, the focus of interventions should be primarily on workflow at the system or organizational level, rather than on the individual provider. This finding is consistent with other studies conducted by members of our research group, which have shown that quality improvement efforts should focus on addressing facility-level performance variations, because of the small amount of variation in performance found at the provider level in comparison to the facility level [23,24]. Current educational efforts provided within the VHA appear to be adequate, but not sufficient by themselves for achieving the desired changes in behavior, and we believe that is likely true of most organized primary care delivery settings in the US.

Finally, strategies for improving participation of physicians in studies of the quality of care need to be identified. Higher participation rates have been observed in minimal-risk, observational studies such as this one that require informed consent without the requirement of written informed consent.

## Competing interests

The author(s) declare that they have no competing interests.

## Authors' contributions

Concept: LAG, LW. Study design: JCL, CPK, LAG, LW. The transcribed notes from interviews, describing in detail the guideline implementation interventions used at each site, were coded by LAG, KPK, LW. Variable definition and analysis: LAG, JCL, SLK, CPK. Results interpretation: LAG, LW, JCL, SLK. Paper preparation: LAG, JCL, LW, CPK, SLK

## Supplementary Material

Additional file 1 Appendix 1: Concordance scoring weights. Theory-derived weighting indicating degree to which each category of intervention is likely to promote practice change among physicians of each type. Click here for file
