# Supplementary material for: An observational study of the effectiveness of practice guideline implementation strategies examined according to physicians' cognitive styles
Source: Implement Sci. 2007 Dec 1;2:41. doi: 10.1186/1748-5908-2-41 (PMC2219964; doi:10.1186/1748-5908-2-41)
Supplement: Additional file 1 — Appendix 1: Concordance scoring weights. Theory-derived weighting indicating degree to which each category of intervention is likely to promote practice change among physicians of each type. [file 1748-5908-2-41-S1.doc]

CONCORDANCE SCORING FOR KNOWLEDGE-BASED STRATEGIES

|  | | | | Seeker | Receptive | Traditional | Pragmatist |
| --- | --- | --- | --- | --- | --- | --- | --- |
| Evidence-Based | Brief | Written | Influential | 4 | 5 | 2 | 4 |
|  |  |  | Non-influential | 4 | 4 | 0 | 3 |
|  |  | Oral | Influential | 4 | 5 | 2 | 5 |
|  |  |  | Non-influential | 4 | 4 | 0 | 4 |
|  | Detailed | Written | Influential | 5 | 4 | 0 | 1 |
|  |  |  | Non-influential | 5 | 3 | -1 | 0 |
|  |  | Oral | Influential | 5 | 4 | 1 | 0 |
|  |  |  | Non-influential | 5 | 3 | -1 | -1 |
| Authority-Based | Brief | Written | Influential | 0 | 1 | 3 | 4 |
|  |  |  | Non-influential | 0 | 0 | 0 | 3 |
|  |  | Oral | Influential | 0 | 1 | 4 | 5 |
|  |  |  | Non-influential | 0 | 0 | 0 | 4 |
|  | Detailed | Written | Influential | 0 | 2 | 4 | 1 |
|  |  |  | Non-influential | 0 | 0 | 1 | 0 |
|  |  | Oral | Influential | 0 | 2 | 5 | 0 |
|  |  |  | Non-influential | 0 | 0 | 2 | -1 |

# CONCORDANCE SCORING FOR BEHAVIOR-BASED STRATEGIES

|  | | | | Seeker | Receptive | Traditional | Pragmatist |
| --- | --- | --- | --- | --- | --- | --- | --- |
| Organiza-tional | Make it easier | Barrier removal | Based on thorough evaluation | 5 | 5 | 5 | 5 |
|  |  |  | Based on casual evaluation | 2 | 2 | 2 | 3 |
|  |  |  | No evaluation | 1 | 1 | 1 | 2 |
|  |  | Assistive mechanisms | | 5 | 5 | 2 | 3 |
|  | Make it harder: set up barriers | | | 1 | 3 | 2 | 5 |
| Motivational | Make it easier through incentives | Individualized | | 3 | 4 | 3 | 5 |
|  |  | Group | | 1 | 2 | 1 | 3 |
|  | Penalties | Individualized | | 0 | 3 | 2 | 4 |
|  |  | Group | | 0 | 1 | 1 | 3 |
|  | Performance  Feedback | Individualized | | 0 | 4 | 4 | 3 |
|  |  | Group | | 0 | 2 | 1 | 1 |
